# EEG Connectivity during Active Emotional Musical Performance

**DOI:** 10.3390/s22114064

**Published:** 2022-05-27

**Authors:** Mahrad Ghodousi, Jachin Edward Pousson, Aleksandras Voicikas, Valdis Bernhofs, Evaldas Pipinis, Povilas Tarailis, Lana Burmistrova, Yuan-Pin Lin, Inga Griškova-Bulanova

**Affiliations:** 1 Department of Neurobiology and Biophysics, Vilnius University, 10257 Vilnius, Lithuania; mahrad.ghodousi@gmc.stud.vu.lt (M.G.); aleksandras.voicikas@gmc.vu.lt (A.V.); evaldas.pipinis@gmc.vu.lt (E.P.); povilas.tarailis@gmc.vu.lt (P.T.); 2 Jāzeps Vītols Latvian Academy of Music, 1050 Riga, Latvia; jachin.edward.pousson@jvlma.lv (J.E.P.); valdis.bernhofs@jvlma.lv (V.B.); lana.burmistrova@jvlma.lv (L.B.); 3 Institute of Medical Science and Technology, National Sun Yat-sen University, Lienhai Road, Kaohsiung 80424, Taiwan; yplin0115@gmail.com; 4 Department of Electrical Engineering, National Sun Yat-sen University, Lienhai Road, Kaohsiung 80424, Taiwan

**Keywords:** music, emotion, active performance, EEG, connectivity

## Abstract

The neural correlates of intentional emotion transfer by the music performer are not well investigated as the present-day research mainly focuses on the assessment of emotions evoked by music. In this study, we aim to determine whether EEG connectivity patterns can reflect differences in information exchange during emotional playing. The EEG data were recorded while subjects were performing a simple piano score with contrasting emotional intentions and evaluated the subjectively experienced success of emotion transfer. The brain connectivity patterns were assessed from the EEG data using the Granger Causality approach. The effective connectivity was analyzed in different frequency bands—delta, theta, alpha, beta, and gamma. The features that (1) were able to discriminate between the neutral baseline and the emotional playing and (2) were shared across conditions, were used for further comparison. The low frequency bands—delta, theta, alpha—showed a limited number of connections (4 to 6) contributing to the discrimination between the emotional playing conditions. In contrast, a dense pattern of connections between regions that was able to discriminate between conditions (30 to 38) was observed in beta and gamma frequency ranges. The current study demonstrates that EEG-based connectivity in beta and gamma frequency ranges can effectively reflect the state of the networks involved in the emotional transfer through musical performance, whereas utility of the low frequency bands (delta, theta, alpha) remains questionable.

## 1. Introduction

The proposed mechanisms of emotion induction by music differ in many aspects including the information focus, cultural impact, dependence on musical structure, evaluative conditioning, and others. These are important from the listener’s perspective; however, these are also implicated in intended emotion transfer while performing. The present-day research mainly focuses on the assessment of emotions evoked by music. The neural correlates of intentional emotion transfer by the music performer, however, are not well investigated. 

McPherson et al. [1] utilizing fMRI discovered that, during the creative expression of emotions through music, emotion-processing areas of the brain are activated in ways that differ from the perception of emotion in music. However, the electroencephalogram (EEG), being a unique real-time brain activity assessment method, appears to be advantageous in the context of music performance allowing more ecologically valid settings. Nevertheless, only few studies related to music improvisation or active playing while collecting and interpreting EEG data [2,3,4]. In our recent study, we investigated spectral properties of EEG activity in musicians while they were instructed to transfer a certain emotion through performance of a predefined simple music score. To our knowledge, it was the first attempt to address the intended emotional communication through an artistic medium using the EEG signal. As the emotion to be communicated by musicians does not necessarily reflect their actual felt emotions in the moment [5,6], subjects self-evaluated their own performances based on how well they felt they expressed the intended emotion within it. 

The modulation of emotional intent via the means of expressive cues in academic music is often written in the score, and executed by the performer. However, in jazz or popular music, these expressive cues are often only tacitly implied and the performer is given room for interpretation. For example, the performer may often seek to perform a unique or characteristic rendition of a familiar song by changing the tempo, groove, dynamic, or articulation to help communicate their emotional intent [7,8]. The leeway and capacity that performers have to use expressive cues differs between musical genres and performance environments. As a result, the live version of a song may greatly vary from the studio recording depending on the situational context. From a musicology perspective, modulation of affective intent via expressive cues executed in performance is considered an inextricable aspect of the process of embodied musical communication, which is often overlooked in brain-imaging studies using a musical framework [9,10]. Music created, experienced, and consumed in everyday life often functions as a means of mood modulation, and a catalyst for social cohesion, coordination or contextual human behavior. These aspects are difficult to replicate in highly controlled settings where the focus may be on the isolation of a particular response [11,12]. To address these issues, and capture the neural activity related to elusive creative process of imbuing music with emotion in performance, our study’s approach attended to maintaining a level of ecological validity. The recordings took place in a room at the music academy in Riga, where musicians are familiar with practicing, performing, and recording. Musicians were informed that the audio recordings of their performances would be evaluated later by a listener group. This knowledge helped performers to associate each session of EEG recording with an ordinary music studio recording session they may experience in their everyday practice. We probed the brain activity patterns that are differentially involved in distinct emotional states by employing the experimental contrast of emotional playing vs. neutral playing. Differences in power of EEG activity were observed between distressed/excited and neutral/depressed/relaxed playing conditions [13].

The integration of different cortical areas is required for both music perception and emotional processing. Several attempts have been made to investigate network connectivity in relation to emotional aspects of music listening. Previous studies targeting at emotion discrimination while listening to music demonstrated that distinct network connectivity and activation patterns of target regions in the brain are present during listening, particularly between the auditory cortex, the reward brain system, and brain regions active during mind wandering [14]. The existing fMRI-based studies showed that clear variations in connectivity for different music pieces are present. Karmonik et al. [15] reported largest activation for processing of self-selected music with emotional attachment or culturally unfamiliar music. Recently, Liu et al. [16] associated emotional ratings of pleasure and arousal with brain activity. In their study, classical music was associated with the highest pleasure rating and deactivation of the corpus callosum while rock music was associated with the highest arousal rating and deactivation of the cingulate gyrus. Pop music, in contrast, activated the bilateral supplementary motor areas and the superior temporal gyrus with moderate pleasure and arousal. Using EEG, Varotto et al. [17] demonstrated that the pleasant music induces an increase in network number of connections, compared with the resting condition, while no changes are caused by the unpleasant stimuli. Shahabi and Moghimi [18] reported a positive association between perceived valence and the frontal inter-hemispheric flow, but a negative correlation with the parietal bilateral connectivity while listening to music. Recently, Mahmood et al. [19] demonstrated that even a short period of listening to music can significantly change the connectivity in the brain.

Importantly, the activation patterns while listening to music may differ in musicians when compared to non-musicians as demonstrated by Alluri et al. [20]: in their study, musicians automatically engaged action-based neural networks (cerebral and cerebellar sensorimotor regions) while listening to music, whereas non-musicians used perception-based networks to process the incoming auditory stream. However, it is not well known to what extent the connectivity differs between states of active emotional performances. With this follow up study, we aim to determine whether connectivity patterns can reflect differences in information exchange during emotionally imbued playing. We assessed brain connectivity patterns from the EEG data while subjects were performing with the emotional intent. We contrasted emotional playing with neutral playing to control over general patterns of motor and sensory activation and expected that observed connectivity patterns are attributable to the emotion-related aspects of the performance.

## 2. Materials and Methods

### 2.1. Participants

Ten musicians (2 males, 8 females; age 19–40 years) were recruited with the criteria that they were experienced piano players with a minimum of 5 years of academic training. For each participant, EEG recording sessions involving a piano-playing task took place over four sessions scheduled on different days. Rīga Stradiņš University Research Ethics Committee approved the study (Nr.6-1/01/59), and all participants provided their written consent.

### 2.2. Experimental Design and Procedure

**Figure 1:**
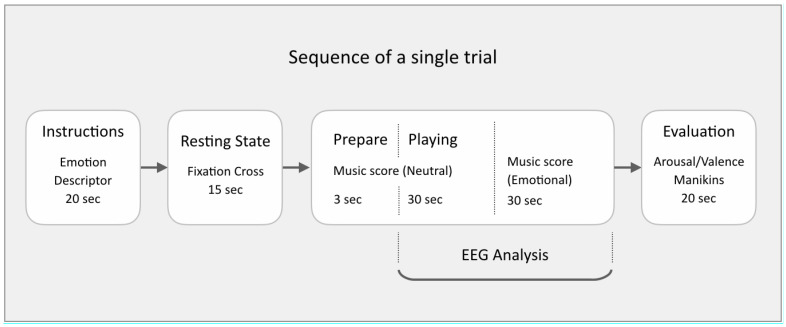
Sequence of a single experimental trial.

Participants were provided with a musical score composed by the author (available in Supplementary Materials), designed to be simple enough for trained pianists to learn quickly and make expressive variation upon. The music used an extended pentatonic scale to circumvent Classical Western functional harmony bias, and presented on two pages. The first page was to be performed mechanically, in tempo, neutral in expression. The music on the second page was a repeat of the first page, but freedom was given to the player to alter their manner of play in order to express one of five emotions based on a 2D valence-arousal model of affective space (distressed, excited, depressed, relaxed, neutral). Participants were encouraged to use any and all expressive cues at their disposal (such as tempo, groove, articulation, embellishment), to make a contrast between the neutral first page and the emotion-imbued second page (except when the second page was also neutral). Each page had duration of 30 s, making the duration of each performance 1 min. The protocol was controlled with the Psychopy stimulus presentation software [21], which presented instructions for each trial in order and randomized the sequence of the five emotions over the course of each recording session. A total of 200 trials were recorded for each participant. See Figure 1 for a schematic representation of a single experimental trial.

All participants were fully briefed on what to expect before their first scheduled recording session. At each first session, participants were given time to familiarise themselves with the recording sequence and emotional descriptors, ensuring their understanding of the piano playing task as well as the self-evaluation step. During each trial, subjects were asked to remain seated at the piano and follow the instructions presented on a laptop screen at eye level. 

**Figure 2:**
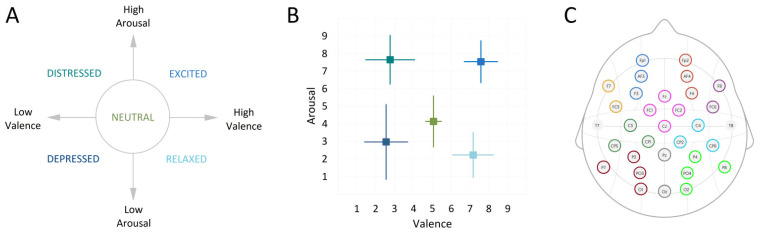
(**A**) Emotion descriptors used for performance instructions; (**B**) mean results of subjective self-assessment on the experienced emotional arousal and valence levels across each experimental trial of all participant (color coding is similar as in A); (**C**) the group of electrodes into regions of interest (ROIs): mid-frontal left (1) and right (2), left (3) and right (4) frontal, centro-parietal left (5) and right (6), parieto-occipital left (7) and right (8), fronto-central (9) and central parieto-occipital (10).

One of the five emotion descriptors was presented for 20 s. Next, a fixation cross was presented for 15 s while recording the resting state. This was followed by the first page of the music score that was presented with a 3 s countdown to start playing. The neutral baseline playing instruction was displayed for 30 s alongside the countdown to the start of emotional playing. This was followed by the second page with the score for emotionally expressive playing lasting 30 s. Participants self-evaluated their own performance on a scale from 1–9, on dimensions of valence (from negative to positive) and arousal (from low to high), with 5 representing neutral on both scales (Figure 2A). Participants were reminded to submit their ratings not based on their actual felt emotions, but, based on how well they felt, their own performance expressed the intended emotion. 

When recording, five trials were grouped into a single run. Ten runs were recorded at each session, with short rests between each run. A total of fifty trials were recorded at each of the four sessions scheduled per participant. Audio from the performance was recorded alongside the EEG, and participants were made aware that these would be evaluated by listeners in future steps.

### 2.3. EEG Acquisition

EEG signals were acquired using an Enobio 32 device, with 32 electrodes placed according to the International 10–20 system. Common Mode Sense (CMS) and Driven Right Leg (DRL) connections were applied to the right earlobe for grounding, while signal quality was monitored within the hardware’s native signal acquisition software Neuroelectrics Instrument Controller v.2.0.11.1 (NIC). The quality index provided within NIC consists of a real-time evaluation of four parameters, namely line noise, main noise, offset, and drift. Data were recorded at a 500 Hz sampling rate with a notch filter applied at 50 Hz to remove power line noise.

### 2.4. EEG Preprocessing

EEG data were prepared for further analysis using an automated Preprocessing Pipeline script in MATLAB and utilizing several functions from the EEGLAB toolbox [22]. First, the Automated Artifact Rejection function in EEGLAB was applied to the raw EEGs to eliminate the bad portions of the data, and the channels that had lost more than 20% of their data were discarded. The data were filtered using the zero-phase bandpass FIR filter between 0.5 to 45 Hz implemented in EEGLAB, and referenced to the mean of T7 and T8 channels. Independent Component Analysis (ICA) and ICLabel plugin in EEGLAB were used to detect and remove the embedded artifacts including muscle activity, eye blinks, eye movements, and heart electrical activity. The 30 s of neutral baseline performance and 30 s of each emotional performance (distressed, excited, depressed, relaxed, and neutral) were extracted resulting in 2000 EEG time series (data from 10 participants across 4 days and 50 piano-playing excerpts per session) for emotional playing (400 segments for each of the emotional instruction, further called observations), and 2000 EEG time series for the corresponding baseline. Emotional playing and baseline time series were treated separately but in the same way. The electrodes were grouped into ten regions of interest (ROI, Figure 2C) and the average for electrodes within the ROI was obtained. The observations that did not contain information on at least one ROI (due to channels removal in previous steps) were excluded from the analysis. To maintain the homogeneity of the final matrix, the minimum number of observations remaining in each emotional condition was equal to 314. The 30-s time series for each of the 10 ROIs were segmented into 3-s epochs starting from 3rd s to 27th s, and the averages of these segmented data were used for further assessments. The dimensions of the final observation matrix thus were equal to 1570 × 10 × 1500 arrays ((314 observations for each of five emotional conditions) × (ROIs) × (3 s × 500 samples per second)). By filtering the separate rows of the observation matrix into different frequency bands for 1–4 Hz (delta), 4–8 Hz (theta), 8–12 Hz (alpha), 12–30 Hz (beta), and 30–45 Hz (gamma), five sub-band observation matrices were obtained for each emotional playing part and corresponding baseline.

### 2.5. EEG Analysis

We focused on the effective connectivity approach that provides information on the direction of information flow in the nerve systems and illustrates complex interactions in the brain regions [23,24,25]. Granger Causality (GC) was utilized as a relatively simple (with low hardware demand) available method of calculating the directed connections that increase the success of implementing a real-time BCMI machine in future research [26].

#### 2.5.1. Granger Causality

GC is a statistical concept of causality which is founded on prediction. According to Granger causality, if a signal X “Granger-causes” a signal Y, then past values of X should provide information that helps to predict Y, whereas the past values in Y alone are not sufficient to predict its future [27]. 

GC is formulated as follows:

Let *y*(*t*) and *x*(*t*) be stationary time series. First, find the proper lagged values of *y*(*t − i*) to include in the univariate autoregression of *y*(*t*) according to (1):

(1) $$y\left(t\right)=\varepsilon \left(t\right)+\sum_{i=1}^{\infty }a(i)\times y(t-i)$$

Then, the autoregression is recalculated by including lagged values of *x*(*t*) as follows (2):

(2) $$y\left(t\right)=\tilde{\varepsilon }\left(t\right)+\sum_{i=1}^{\infty }a(i)\times y(t-i)+\sum_{j=1}^{\infty }b(i)\times x(t-j)$$

where *a*(*i*) and *b*(*j*) are regressive coefficients and $\tilde{\varepsilon }\left(t\right)$ is the prediction error calculated by considering the effect of lagged values of *x*(*t*) on predicting the *y*(*t*), and $\varepsilon \left(t\right)$ is the prediction error of *y*(*t*) calculated without using *x*(*t*). Therefore, if the variance of $\tilde{\varepsilon }\left(t\right)$ is smaller than that variance of $\varepsilon \left(t\right)$, then the GC will be 1 and *x*(*t*) ‘’Granger-causes’’ *y*(*t*); if the variance of $\tilde{\varepsilon }\left(t\right)$ is larger than that variance of $\varepsilon \left(t\right)$, then GC value will be 0 and *x*(*t*) does not ‘’Granger-cause’’ *y*(*t*).

#### 2.5.2. Feature Extraction

For all observations, the GC was calculated between each pair of the ROIs, and 10 × 10 connectivity matrices were created, where the array (i, j) illustrates the GC value between channels i, j. The connectivity matrices were further transformed into a 100-element row by placing their 10 × 10 arrays sequentially next to each other, resulting in the observation matrices of 1570 × 100 arrays instead of 1570 × 10 × 1500 [23].

#### 2.5.3. Feature Selection

To reduce the number of features in the emotional and baseline matrices, the following steps were taken. First, it was expected that the neutral baseline EEG and emotional playing EEG would be different, thus a two-sided Student *t*-test was applied to the data in each column in the feature matrix belonging to one emotional category and the same column in the baseline matrix. The null hypothesis (both data are drawn from the same distribution) was rejected with *p*-values < 0.05. Since no significant differences between the ‘neutral baseline EEGs’ and the ‘EEG recorded during the expressing neutral emotions’ were expected, this step was ignored for 314 observations of neutral playing. The remaining features that (1) were able to discriminate between the neutral baseline and the emotional playing, and (2) were shared across conditions, were used to create new feature matrices.

Furthermore, the one-way MANOVA [28] analysis implemented in MATLAB was performed to identify the features that were able to discriminate between conditions. The components (the linear combination of the features) were created based on the canonical correlation analysis. For each frequency band, four components were retained with *p*-values < 0.05, and the statistical outcome is presented in Supplementary Materials. The features that contributed to the selected components were chosen as a set of final features.

#### 2.5.4. Graph Quantification

After selecting the features, the matrices of observations were averaged to obtain a single average connectivity matrix per condition with arrays describing the strength of the established connections with values close to 0 indicating a weak connection and values close to 1 indicating a strong connection. Since the connectivity matrices are similar to graphs which contain ROIs as nodes and connections as edges, we applied graph quantifying techniques to statistically evaluate the patterns [18,29]. The degree of nodes referring to the number of outputs from each node were calculated for 1570 connectivity matrices, and, for each ROI (node), one-way ANOVA was performed on 10 pairs of emotional states (C(5,2) = 10). By considering 10 nodes and 10 pairs, 100 *p*-values and 100 of Cohen’s d effect sizes were obtained.

## 3. Results

As expected, the tasks of emotional and neutral playing differed considerably in terms of arousal and valence levels with respect to the state of the intended emotion transfer. The means and standard deviations of subjective evaluation on the scales of valence and arousal are plotted in Figure 2B.

Differences in connectivity were expected to be observed in performers for different emotional instructions. However, the number of initial extracted features being very high made results not possible to comprehend. Thus, a further feature reduction was performed and only the features that were able to discriminate between the neutral baseline and the emotional playing and were shared across conditions were utilized further.

The number of selected features that were able to discriminate between conditions is presented in Table 1. 

**Figure 3:**
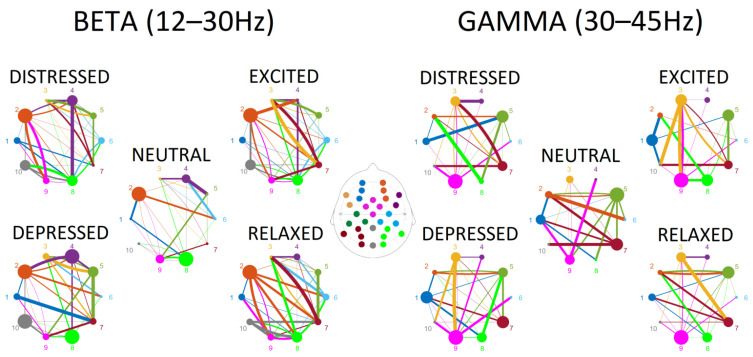
The connectivity plots in the beta and gamma frequency ranges for five experimental conditions—neutral, depressed, distressed, excited, and relaxed performance instructions. Colors represent ROIs (also coded as numbers: mid-frontal left (1) and right (2), fronto-lateral left (3) and right (4), centro-parietal left (5) and right (6), parieto-occipital left (7) and right (8), fronto-central (9) and central parieto-occipital (10)) and corresponding connections from each ROI. The size of the circle reflects the degrees of nodes, and the thickness of the lines reflects the strength of the connection.

Surprisingly, for the low frequency bands—delta, theta, alpha—a limited number of connections (4 to 6) contributed to the discrimination between the emotional playing conditions. In contrast, a dense pattern of connections between regions that was able to discriminate between conditions was observed in beta and gamma frequency ranges (30 to 38). Tables containing statistical outputs and visualization of connectivity for all frequency bands are provided in the Supplementary Materials. The connectivity patterns for beta and gamma ranges are plotted in Figure 3, where outflow connections are color-coded in the same way as the ROIs, and the degrees of node are represented by the size of the node, and strength of the connections is reflected by the thickness of the lines.

**Figure 4:**
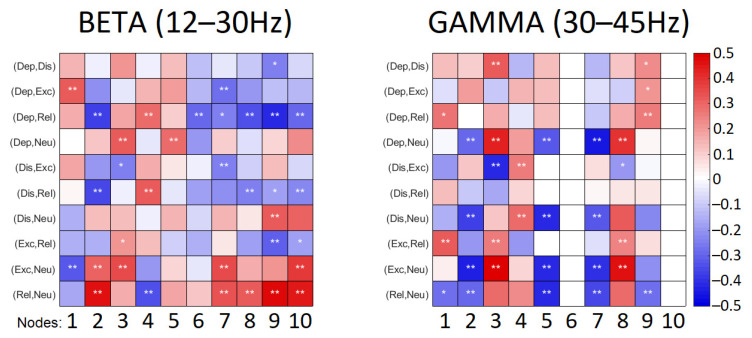
Results of comparison of the degrees of nodes: color codes the effect sizes corresponding to the difference in the mean of the groups and *p* values are marked with *: * denotes *p* < 0.05, ** denotes *p* < 0.01. Dep—depressed, Dis—distressed, Exc—excited, Rel—relaxed, Neu—neutral. ROIs are coded in numbers: mid-frontal left (1) and right (2), fronto-lateral left (3) and right (4), centro-parietal left (5) and right (6), parieto-occipital left (7) and right (8), fronto-central (9) and central parieto-occipital (10).

As the strength of the connections identified in frequency bands can either increase or decrease reflecting in degrees of nodes, the degrees of nodes for all emotional conditions were followed by a pairwise comparison between emotional instructions. The statistical outcomes of this comparison for beta and gamma ranges are illustrated in Figure 4.

Connections observed in the beta range were more abundant in emotional playing conditions in comparison to neutral playing (Figure 3). The low valence conditions—distressed and depressed—were characterized by somewhat reduced node densities in fronto-central, right centro-parietal, right and left parieto-occipital regions. This was especially pronounced when depressed state was contrasted with relaxed state (Figure 4). The high valence states—excited and relaxed—were characterized by denser right fronto-lateral, fronto-central and parieto-occipital nodes when compared to the neutral condition; this effect was stronger for relaxed-neutral contrast. Moreover, the right mid-frontal node was somewhat more connected to the left and right parieto-occipital regions, and connections with the right fronto-lateral node were reduced.

Connections in the gamma range, although abundant, were not easily discriminating based on either valence or arousal (Figure 3). The right mid-frontal, left centro-parietal and parieto-occipital nodes showed reduced degrees of nodes in emotional playing conditions when compared to neutral playing. However, an opposite effect was observed for the left fronto-lateral and right-parieto-occipital regions, where the node density was higher during emotional performance. In the low arousal states, the left mid-frontal node was connected to both the right and left parieto-occipital nodes, but not this was not seen in the high arousal states. In high arousal conditions, the left parieto-occipital node was somewhat better connected to the left fronto-lateral and central parieto-occipital nodes. Surprisingly, the right centro-parietal and parieto-occipital node showed a very limited connectivity to other nodes in the gamma range (Figure 4).

## 4. Discussion

The aim of the present study was to examine the organization of networks during music performance with the intention to transfer emotion. The EEG responses were collected and the patterns of connectivity were estimated using Granger causality approach. The effective (signal flow) connectivity was analyzed in different frequency bands, i.e., delta, theta, alpha, beta, and gamma. As the connectivity patterns are complex and difficult to evaluate, a set of features able to discriminate between conditions was identified first and analyzed further.

The analysis of the spectral properties of EEG reported in our previous study [13] on the same dataset suggested differences between high-arousal and low-arousal conditions to be reflected in elevated frontal delta and theta activity and signs of increased frontal and posterior beta and gamma. Surprisingly, the connections within the low (delta, theta and alpha) frequency bands did not show a potential to distinguish among emotional playing tasks. This could indicate that the similar connection pattern was present in all emotional playing conditions in the low frequencies. In contrast, the activity in higher frequencies—beta and gamma ranges—demonstrated a dense connection pattern discriminating between emotional performance conditions. The observed effect matches the results by Dolan et al. [30], who, using brain entropy and signal complexity, demonstrated that the prepared performance was associated with the activity in low frequencies (delta, theta and alpha bands), while the improvisation required activity at higher frequencies (beta and gamma).

Improvisatory behavior in music was previously related to network of prefrontal brain regions commonly linked to the presupplementary motor area, medial prefrontal cortex, inferior frontal gyrus, dorsolateral prefrontal cortex, and dorsal premotor cortex [31]. We showed that connections from frontal regions to all other regions were present and expressed. This pattern might reflect the activation of anterior brain regions contributing to musical structure building when performing [32], emotional processing where the prefrontal region plays the most important role and interacts with almost all other regions [33], and also executive functioning [34].

Surprisingly, however, in the gamma range, the right mid-frontal node displayed reduced density during emotional performance when compared to neutral playing. This observation could be partly related to the results by Pinho et al. [35]. The authors showed a decrease in dorso-lateral prefrontal cortex activity in professional pianists improvising based on specific emotional cues (happy/fearful) but an increase in activity in the same region when the improvisation was based on specific pitch sets. For the left lateral frontal region, however, an opposite effect was observed—the node density was higher during the emotional performance in gamma and, partly, in beta ranges. Previously, Bhattacharya et al. [36] demonstrated the leftward dominance for the degree of gamma band synchrony in musicians while they were listening to music than in non-musicians and attributed this effect to the manifestation of musical memory. Correspondingly, in the beta frequency band, the predominant activation in the left hemisphere along with the inter-hemispheric integration between the frontal right and parietal left region during improvisation were observed by Rosen et al. [2] in professional musicians but not in amateur musicians [4]. Finally, Kaneda et al. [37] indicated that the left inferior frontal activation might contribute to generation of emotions by semantic elaboration and regulation through reappraisal. Taking into account the setup of the current experiment, where subjects were asked to reflect certain emotion and evaluate the success, it is well likely that semantic and reappraisal-related processes were activated during the performance. Thus, the denser connections in the high frequency bands, capable of showing the most significant differences among the positive, neutral, and negative emotional states [38], may indicate the mediation of information transmission during the processing of emotion-related activities [39] and the cognitive aspects of the active performance [36] on the one hand. On the other hand, it suggests that professional training in music allows a distinct context-sensitive functional connectivity between multiple cortical regions to occur during listening to music [36], and potentially it depends on the level of experience [2]. 

Investigating the neural underpinnings of active emotional music performance promotes a better understanding of human creative processes and capabilities. Notably, the connectivity measures seem to reflect different aspects of emotional performance than classical spectral EEG measures. The different approaches allow for identifying neural signatures potentially applicable in Brain–Computer Music Interface (BCMI) systems designed for supporting embodied music performance, or neurofeedback contexts where a user may learn to control musical parameters via their online EEG signal. However, the study design, as utilized in the present study, is complex and repetitive, allowing unique data collection but resulting in a small sample size. This prevented the evaluation of the effects of individual factors such as gender, age or musical experience. Future works should test the suitability of the extracted parameter for BCMI use and selection of effective classification approaches [40].

## 5. Conclusions

The current study demonstrates that EEG-based connectivity in beta and gamma frequency ranges can effectively reflect the state of the networks involved in the emotional transfer through musical performance, whereas utility of the low frequency bands (delta, theta, alpha) remains questionable.

## Figures and Tables

**Table 1:** Number of features retained after feature extraction steps.

	Student *t*-Test (Number of Features)	MANOVA (Number of Features)
Delta	9	6
Theta	11	6
Alpha	8	4
Beta	46	38
Gamma	45	30

## Data Availability

The data presented in this study are available on request from the corresponding author. The data are not publicly available due to privacy restrictions.

## Supplementary Materials

The following supporting information can be downloaded at: https://www.mdpi.com/article/10.3390/s22114064/s1, Figure S1: Musical score; Figure S2: Connectivity in delta, theta and alpha bands; Table S1: MANOVA outcomes; Table S2: Statistical outcomes on pairwise comparison for degrees of nodes.
