# Supplementary material for: EEG Connectivity during Active Emotional Musical Performance
Source: Sensors (Basel). 2022 May 27;22(11):4064. doi: 10.3390/s22114064 (PMC9185252; doi:10.3390/s22114064)
Supplement: Supplementary file 1 [file sensors-22-04064-s001.zip › Table S1.pdf]

DELTA (1-4Hz)

	F (4,1565)	P values	Wilk's Lambda	Chi-square	Eta^2
1st comp	44	0.0000	0.811	326	0.100
2nd comp	24	0.0000	0.901	161	0.059
3rd comp	11	0.0000	0.958	65	0.028
4th comp	5.3	0.0008	0.986	21	0.013

THETA (4-8Hz)

	F (4,1565)	P values	Wilk's Lambda	Chi-square	Eta^2
1st comp	35	0.0000	0.801	346	0.082
2nd comp	23	0.0000	0.873	211	0.055
3rd comp	18	0.0000	0.924	123	0.044
4th comp	13	0.0000	0.967	52	0.032

ALPHA (8-12Hz)

	F (4,1565)	P values	Wilk's Lambda	Chi-square	Eta^2
1st comp	38	0.0000	0.844	263	0.081
2nd comp	15	0.0000	0.928	116	0.042
3rd comp	12	0.0000	0.963	58	0.035
4th comp	2.5	0.03	0.993	10	0.006

BETA (12-30Hz)

	F (4,1565)	P values	Wilk's Lambda	Chi-square	Eta^2
1st comp	231	0.0000	0.231	2219	0.37
2nd comp	175	0.0000	0.368	1515	0.31
3rd comp	161	0.0000	0.533	952	0.29
4th comp	127	0.0000	0.754	427	0.25

GAMMA (30-45Hz)

	F (4,1565)	P values	Wilk's Lambda	Chi-square	Eta^2
1st comp	246	0.0000	0.244	2138	0.37
2nd comp	170	0.0000	0.397	1397	0.30
3rd comp	130	0.0000	0.571	849	0.25
4th comp	122	0.0000	0.760	414	0.24
