# Supplementary material for: EEG Connectivity during Active Emotional Musical Performance
Source: Sensors (Basel). 2022 May 27;22(11):4064. doi: 10.3390/s22114064 (PMC9185252; doi:10.3390/s22114064)
Supplement: Supplementary file 1 [file sensors-22-04064-s001.zip › Table S2.pdf]

Node Degree, Delta, P values										
Nodes / Pairs	Node1	Node2	Node3	Node4	Node5	Node6	Node7	Node8	Node9	Node10
(Dep, Dis)	1	1	0	1	1	1	1	1	1	1
(Dep, Exc)	1	1	0.989	1	1	1	1	1	0	1
(Dep, Rel)	1	1	0.989	1	1	1	1	1	1	1
(Dep, Neu)	1	1	0.989	1	1	1	1	1	1	0
(Dis, Exc)	1	1	0	1	1	1	1	1	0	1
(Dis, Rel)	1	1	0	1	1	1	1	1	1	1
(Dis, Neu)	1	1	0	1	1	1	1	1	1	0
(Exc, Rel)	1	1	1	1	1	1	1	1	0	1
(Exc, Neu)	1	1	1	1	1	1	1	1	0	0
(Rel, Neu)	1	1	1	1	1	1	1	1	1	0

Node Degree, Delta, Effect Size										
Nodes / Pairs	Node1	Node2	Node3	Node4	Node5	Node6	Node7	Node8	Node9	Node10
(Dep, Dis)	0	0	-0.219	0	0	0	0	0	0	0
(Dep, Exc)	0	0	0.08	0	0	0	0	0	-0.256	0
(Dep, Rel)	0	0	0.08	0	0	0	0	0	0	0
(Dep, Neu)	0	0	0.08	0	0	0	0	0	0	-0.256
(Dis, Exc)	0	0	0.256	0	0	0	0	0	-0.256	0
(Dis, Rel)	0	0	0.256	0	0	0	0	0	0	0
(Dis, Neu)	0	0	0.256	0	0	0	0	0	0	-0.256
(Exc, Rel)	0	0	0	0	0	0	0	0	0.256	0
(Exc, Neu)	0	0	0	0	0	0	0	0	0.256	-0.256
(Rel, Neu)	0	0	0	0	0	0	0	0	0	-0.256

Node Degree, Theta, P values										
Nodes / Pairs	Node1	Node2	Node3	Node4	Node5	Node6	Node7	Node8	Node9	Node10
(Dep, Dis)	1	1	1	1	1	1	1	1	1	1
(Dep, Exc)	1	1	1	1	0	0.888	1	1	1	0
(Dep, Rel)	1	1	1	1	0.672	0	1	1	1	1
(Dep, Neu)	1	1	1	1	1	1	1	1	1	1
(Dis, Exc)	1	1	1	1	0	0.888	1	1	1	0
(Dis, Rel)	1	1	1	1	0.672	0	1	1	1	1
(Dis, Neu)	1	1	1	1	1	1	1	1	1	1
(Exc, Rel)	1	1	1	1	0.016	0.002	1	1	1	0
(Exc, Neu)	1	1	1	1	0	0.888	1	1	1	0
(Rel, Neu)	1	1	1	1	0.672	0	1	1	1	1

Node Degree, Theta, Effect Size										
Nodes / Pairs	Node1	Node2	Node3	Node4	Node5	Node6	Node7	Node8	Node9	Node10
(Dep, Dis)	0	0	0	0	0	0	0	0	0	0

(Dep, Exc)	0	0	0	0	-0.256	-0.113	0	0	0	-0.256
(Dep, Rel)	0	0	0	0	-0.139	-0.256	0	0	0	0
(Dep, Neu)	0	0	0	0	0	0	0	0	0	0
(Dis, Exc)	0	0	0	0	-0.256	-0.113	0	0	0	-0.256
(Dis, Rel)	0	0	0	0	-0.139	-0.256	0	0	0	0
(Dis, Neu)	0	0	0	0	0	0	0	0	0	0
(Exc, Rel)	0	0	0	0	0.157	-0.187	0	0	0	0.256
(Exc, Neu)	0	0	0	0	0.256	0.113	0	0	0	0.256
(Rel, Neu)	0	0	0	0	0.139	0.256	0	0	0	0

Node Degree, Alpha, P values

Nodes / Pairs	Node1	Node2	Node3	Node4	Node5	Node6	Node7	Node8	Node9	Node10
(Dep, Dis)	1	1	1	1	1	1	1	1	1	1
(Dep, Exc)	1	1	1	1	1	0.003	1	1	1	1
(Dep, Rel)	1	1	1	1	1	0.003	1	1	1	1
(Dep, Neu)	1	1	1	1	1	0.003	1	1	1	1
(Dis, Exc)	1	1	1	1	1	0.003	1	1	1	1
(Dis, Rel)	1	1	1	1	1	0.003	1	1	1	1
(Dis, Neu)	1	1	1	1	1	0.003	1	1	1	1
(Exc, Rel)	1	1	1	1	1	1	1	1	1	1
(Exc, Neu)	1	1	1	1	1	1	1	1	1	1
(Rel, Neu)	1	1	1	1	1	1	1	1	1	1

Node Degree, Alpha, Effect Size

Nodes / Pairs	Node1	Node2	Node3	Node4	Node5	Node6	Node7	Node8	Node9	Node10
(Dep, Dis)	0	0	0	0	0	0	0	0	0	0
(Dep, Exc)	0	0	0	0	0	0.256	0	0	0	0
(Dep, Rel)	0	0	0	0	0	0.256	0	0	0	0
(Dep, Neu)	0	0	0	0	0	0.256	0	0	0	0
(Dis, Exc)	0	0	0	0	0	0.256	0	0	0	0
(Dis, Rel)	0	0	0	0	0	0.256	0	0	0	0
(Dis, Neu)	0	0	0	0	0	0.256	0	0	0	0
(Exc, Rel)	0	0	0	0	0	0	0	0	0	0
(Exc, Neu)	0	0	0	0	0	0	0	0	0	0
(Rel, Neu)	0	0	0	0	0	0	0	0	0	0

Node Degree, Beta, P values

Nodes / Pairs	Node1	Node2	Node3	Node4	Node5	Node6	Node7	Node8	Node9	Node10
(Dep, Dis)	0.256	1	0.054	0.999	0.306	0.78	0.994	0.733	0.03	0.947
(Dep, Exc)	0.001	0.061	0.987	0.293	0.069	0.603	0.001	0.166	0.754	0.492
(Dep, Rel)	0.135	0	0.126	0.006	0.557	0.003	0.024	0	0	0
(Dep, Neu)	1	0.826	0.001	0.989	0.003	0.26	0.878	0.981	0.96	0.342
(Dis, Exc)	0.336	0.104	0.012	0.184	0.96	0.999	0.004	0.852	0.429	0.909

(Dis, Rel)	0.998	0	0.997	0.002	0.994	0.104	0.076	0.003	0.03	0.002
(Dis, Neu)	0.336	0.706	0.763	0.999	0.468	0.911	0.658	0.963	0.003	0.071
(Exc, Rel)	0.527	0.104	0.034	0.589	0.808	0.197	0.878	0.075	0	0.036
(Exc, Neu)	0.002	0.002	0	0.107	0.873	0.979	0	0.447	0.329	0.005
(Rel, Neu)	0.189	0	0.551	0.001	0.239	0.51	0.001	0	0	0

Node Degree, Beta, Effect Size

Nodes / Pairs	Node1	Node2	Node3	Node4	Node5	Node6	Node7	Node8	Node9	Node10
(Dep, Dis)	0.155	-0.021	0.205	-0.018	0.138	-0.115	-0.04	-0.103	-0.244	-0.068
(Dep, Exc)	0.324	-0.209	-0.032	0.151	0.197	-0.132	-0.274	-0.196	-0.119	-0.14
(Dep, Rel)	0.171	-0.365	0.175	0.295	0.108	-0.285	-0.243	-0.34	-0.408	-0.282
(Dep, Neu)	0.011	0.115	0.323	-0.036	0.284	-0.192	0.106	-0.054	0.093	0.227
(Dis, Exc)	0.185	-0.191	-0.236	0.17	0.056	-0.023	-0.242	-0.083	0.13	-0.075
(Dis, Rel)	0.026	-0.347	-0.031	0.318	-0.032	-0.175	-0.205	-0.242	-0.176	-0.234
(Dis, Neu)	-0.144	0.136	0.13	-0.018	0.143	-0.071	0.145	0.06	0.322	0.311
(Exc, Rel)	-0.143	-0.154	0.207	0.137	-0.089	-0.147	0.059	-0.18	-0.302	-0.175
(Exc, Neu)	-0.314	0.312	0.352	-0.188	0.09	-0.045	0.357	0.159	0.205	0.379
(Rel, Neu)	-0.16	0.464	0.16	-0.335	0.179	0.11	0.342	0.316	0.477	0.445

Node Degree, Gamma, P values

Nodes / Pairs	Node1	Node2	Node3	Node4	Node5	Node6	Node7	Node8	Node9	Node10
(Dep, Dis)	0.421	0.887	0	0.211	0.684	1	0.618	0.478	0.026	1
(Dep, Exc)	0.936	0.454	0.52	0.553	0.684	1	0.98	0.711	0.038	1
(Dep, Rel)	0.025	1	0.153	0.974	0.684	1	0.789	0.19	0.003	1
(Dep, Neu)	1	0	0	0.363	0	1	0	0	0.999	1
(Dis, Exc)	0.09	0.947	0	0.003	1	1	0.915	0.031	1	1
(Dis, Rel)	0.721	0.887	0.345	0.553	1	1	0.999	0.983	0.968	1
(Dis, Neu)	0.33	0	0.914	0.001	0	1	0	0.051	0.055	1
(Exc, Rel)	0.002	0.454	0.001	0.211	1	1	0.98	0.005	0.939	1
(Exc, Neu)	0.971	0	0	0.998	0	1	0	0	0.077	1
(Rel, Neu)	0.015	0	0.054	0.109	0	1	0	0.19	0.008	1

Node Degree, Gamma, Effect Size

Nodes / Pairs	Node1	Node2	Node3	Node4	Node5	Node6	Node7	Node8	Node9	Node10
(Dep, Dis)	0.139	0.098	0.321	-0.13	0.13	0	-0.13	0.119	0.219	0
(Dep, Exc)	-0.055	0.202	-0.097	0.152	0.127	0	-0.056	-0.079	0.206	0
(Dep, Rel)	0.266	0	0.163	-0.043	0.127	0	-0.107	0.165	0.26	0
(Dep, Neu)	-0.011	-0.293	0.43	0.197	-0.314	0	-0.445	0.406	0.017	0
(Dis, Exc)	-0.192	0.114	-0.42	0.261	0	0	0.075	-0.196	-0.013	0
(Dis, Rel)	0.133	-0.098	-0.17	0.089	0	0	0.024	0.047	0.062	0
(Dis, Neu)	-0.15	-0.363	0.114	0.293	-0.413	0	-0.322	0.316	-0.223	0
(Exc, Rel)	0.316	-0.202	0.262	-0.188	0	0	-0.051	0.241	0.072	0
(Exc, Neu)	0.043	-0.426	0.529	0.08	-0.409	0	-0.393	0.467	-0.207	0
(Rel, Neu)	-0.276	-0.293	0.29	0.228	-0.409	0	-0.345	0.281	-0.268	0
