# Supplementary figures and images for: EEG Connectivity during Active Emotional Musical Performance
Source: Sensors (Basel). 2022 May 27;22(11):4064. doi: 10.3390/s22114064 (PMC9185252; doi:10.3390/s22114064)

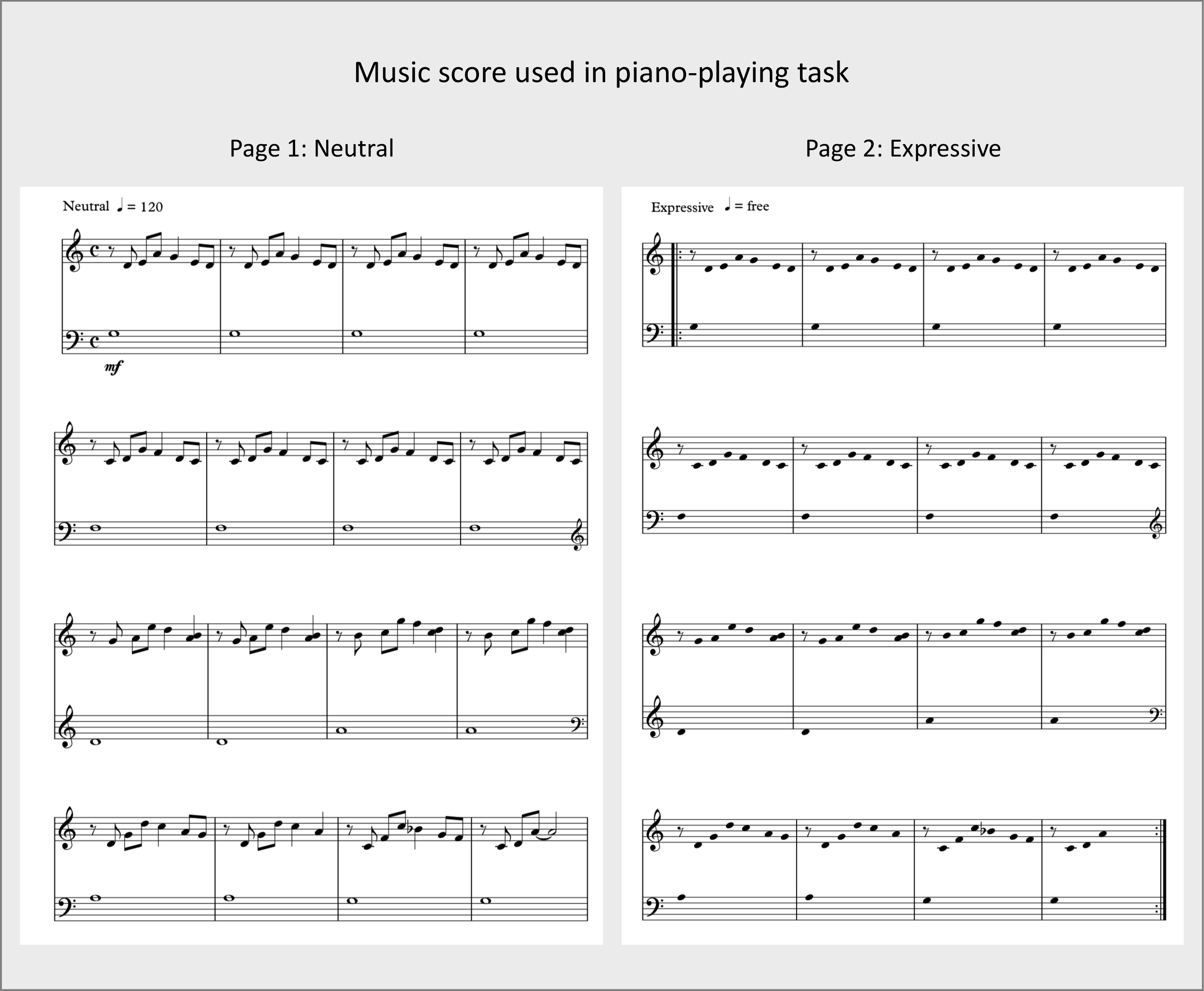

Supplement: Supplementary file 1 [file sensors-22-04064-s001.zip › Figure S1.jpg]

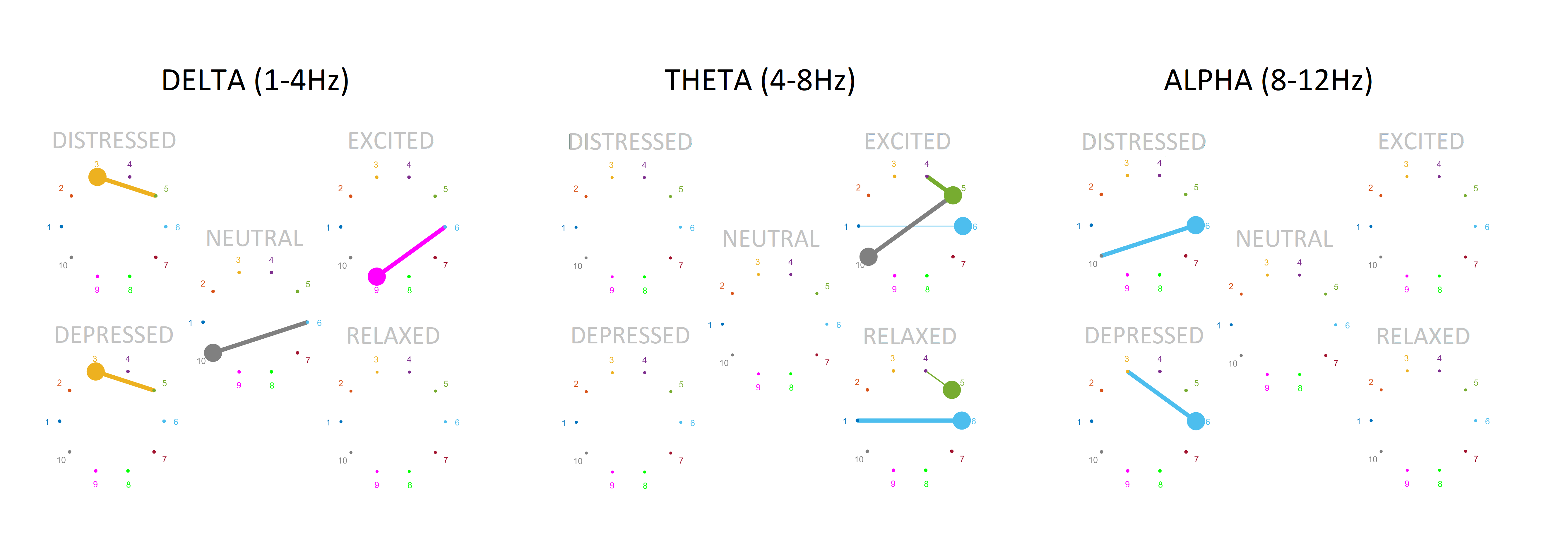

Supplement: Supplementary file 1 [file sensors-22-04064-s001.zip › Figure S2.png]
